# Identifying Corneal Infections in Formalin-Fixed Specimens Using Next Generation Sequencing

**DOI:** 10.1167/iovs.17-21617

**Published:** 2018-01

**Authors:** Zhigang Li, Florian P. Breitwieser, Jennifer Lu, Albert S. Jun, Laura Asnaghi, Steven L. Salzberg, Charles G. Eberhart

**Affiliations:** 1 Department of Ophthalmology, First Affiliated Hospital, Zhengzhou University, Zhengzhou, China; 2 Department of Pathology, Johns Hopkins University, Baltimore, Maryland, United States; 3 Center for Computational Biology, McKusick-Nathans Institute of Genetic Medicine, Johns Hopkins University School of Medicine, Baltimore, Maryland, United States; 4 Department of Biomedical Engineering, Johns Hopkins University, Baltimore, Maryland, United States; 5 Department of Ophthalmology, Johns Hopkins University, Baltimore, Maryland, United States; 6 Departments of Computer Science and Biostatistics, Johns Hopkins University, Baltimore, Maryland, United States

**Keywords:** metagenomics, fungal keratitis, *Acanthamoeba*

## Abstract

**Purpose:**

We test the ability of next-generation sequencing, combined with computational analysis, to identify a range of organisms causing infectious keratitis.

**Methods:**

This retrospective study evaluated 16 cases of infectious keratitis and four control corneas in formalin-fixed tissues from the pathology laboratory. Infectious cases also were analyzed in the microbiology laboratory using culture, polymerase chain reaction, and direct staining. Classified sequence reads were analyzed with two different metagenomics classification engines, Kraken and Centrifuge, and visualized using the Pavian software tool.

**Results:**

Sequencing generated 20 to 46 million reads per sample. On average, 96% of the reads were classified as human, 0.3% corresponded to known vectors or contaminant sequences, 1.7% represented microbial sequences, and 2.4% could not be classified. The two computational strategies successfully identified the fungal, bacterial, and amoebal pathogens in most patients, including all four bacterial and mycobacterial cases, five of six fungal cases, three of three *Acanthamoeba* cases, and one of three herpetic keratitis cases. In several cases, additional potential pathogens also were identified. In one case with cytomegalovirus identified by Kraken and Centrifuge, the virus was confirmed by direct testing, while two where *Staphylococcus aureus* or cytomegalovirus were identified by Centrifuge but not Kraken could not be confirmed. Confirmation was not attempted for an additional three potential pathogens identified by Kraken and 11 identified by Centrifuge.

**Conclusions:**

Next generation sequencing combined with computational analysis can identify a wide range of pathogens in formalin-fixed corneal specimens, with potential applications in clinical diagnostics and research.

Corneal infection remains a significant health problem worldwide.^1^ Prevalence varies widely between studies, but the World Health Organization (WHO) has estimated that corneal ulcers lead to over one million cases of monocular blindness each year.^2^ While diagnostic and therapeutic techniques have improved, increased precision still is needed in our treatment of infectious keratitis patients, and has the potential to decrease morbidity significantly.

Advanced sequencing strategies hold great promise for the diagnosis of infections in the eye and other tissues.^3^ The dramatically increased speed and reduced cost of next generation sequencing (NGS), coupled with new bioinformatics techniques, has made it possible to identify directly and classify nonhuman DNA and RNA sequences in complex specimens. NGS works well with short nucleic acid sequences; thus, it can be used easily to analyze fragmented DNA and RNA extracted from standard formalin-fixed paraffin-embedded (FFPE) clinical specimens left over from routine surgical pathology studies. Such metagenomic analyses, in which all the DNA in a specimen is sequenced without targeting any particular species, have the potential to replace a range of culture, microscopic, and PCR tests with a single sequencing-based approach that could identify any type of organism, including mixtures of pathogens.

We recently reported using NGS, in combination with a new computational analysis pipeline, to identify pathogenic organisms in central nervous system biopsies.^4^ In the current retrospective case series, we sought to apply a similar approach to the analysis of corneal infections, including eukaryotic species that we had not encountered in the brain. Infectious keratitis cases for which an infectious pathogen had been identified previously by culture, microscopic analysis, or a combination of techniques, were analyzed by NGS to provide initial data on the ability of this approach to detect a range of infectious agents.

## Methods

### Clinical Material

Pathology records were searched retrospectively for cases with a microscopic diagnosis of bacterial, viral, fungal or *Acanthamoeba* keratitis. Those with additional confirmation of infectious etiology based on culture, PCR, or direct examination in the microbiology laboratory at Johns Hopkins Hospital were used when possible (Table 1). The infected specimens analyzed included 14 penetrating keratoplasties, an enucleation due to perforation of a corneal ulcer, and one small limbal biopsy. Initial controls included three penetrating keratoplasty specimens and one posterior corneal lamella, all from failed grafts with no clinical or histopathologic suspicion of infection. These cases were not consecutive, and some were several years old; all were processed routinely, with formalin fixation and paraffin embedding. A second set of controls included three anterior lamellar keratoplasty specimens in which sterile tissue from the operating room was divided, with one third frozen for DNA extraction, and the remaining portion sent for diagnosis after routine formalin fixation and processing. All tissue samples were anonymized after initial abstraction of basic demographic and clinical data, and the study was Health Insurance Portability and Accountability Act (HIPAA)-compliant and performed with approval of the Johns Hopkins Institutional Review Board.

**Table 1:**
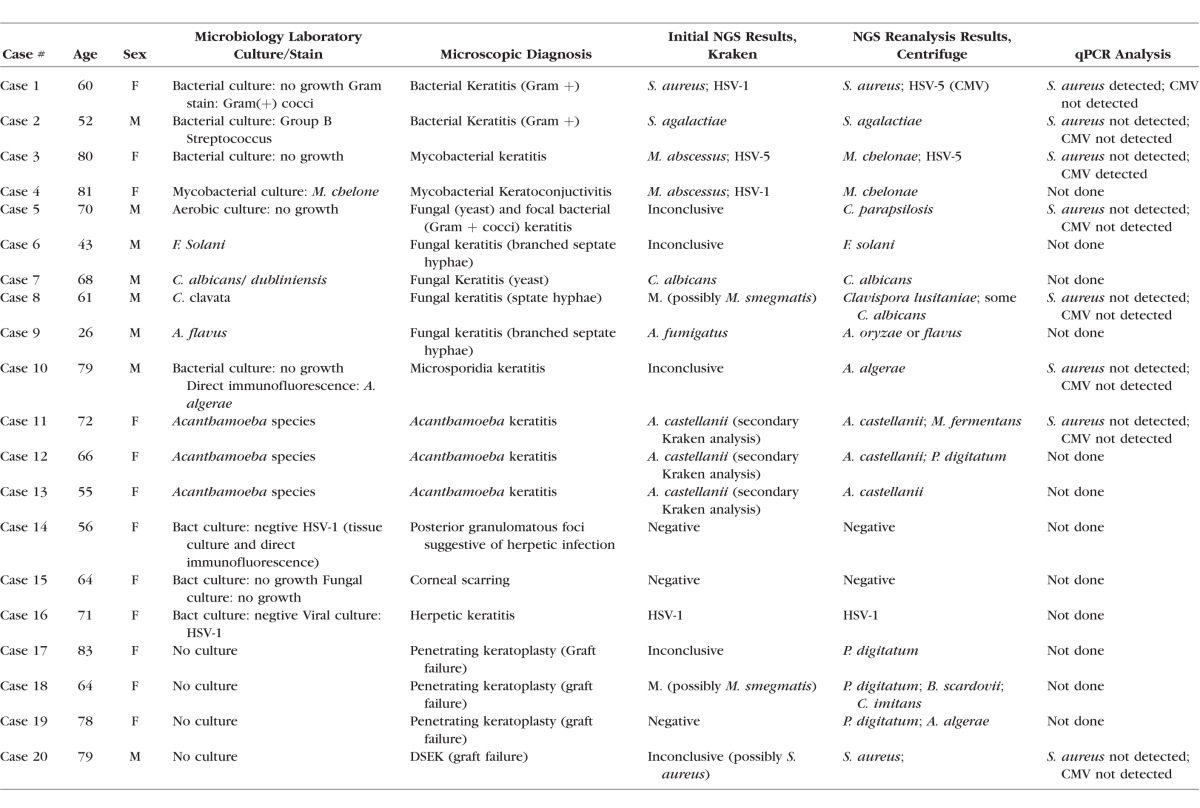
Summary of Clinical, Microscopic, and Sequencing Results

### DNA Sequencing and Computational Analysis

For each paraffin embedded case, 10 sections 10 μm thick were cut into sterile tubes using fresh blades for each sample, while in the three sterile controls, the entire frozen tissue was used. DNA isolation was performed in the Johns Hopkins Sequencing Core using a Qiagen (Hilden, German) QiaAmp FFPE DNA isolation kit with modification. Briefly, the scrolls were deparaffined with 1 mL of Xylene with vigorous vortexing, followed by centrifugation in a microfuge at maximum speed for two minutes. The pellet was washed with 100% ethanol and with centrifugation at maximum speed for two minutes. The pellet was allowed to dry at room temperature for 10 minutes with lid open to allow all ethanol to evaporate. The pellet then was resuspended in 60 μL 1X TE buffer and mixed with 20 μL 1U/μL lyticase and incubated in 37°C oven with constant rotation at 10 revolutions per minute (rpm) for 30 minutes. After incubation, 180 μL ATL and 20 μL Proteinase K was added to the sample and mixed by vortexing. The sample then was incubated at 56°C for 1 hour and followed at 90°C for 1 hour. Due to the low yield of the DNA sample, the DNA concentration was measured using the Qubit dsDNA high sensitivity assay. No other QC was performed. For library preparation, 10 ng DNA was used as input and the library was prepared using Nugen Ultralow library preparation protocol (ver. 2) following the manufacturer's recommended procedure.

Cases were processed in two groups of 10 samples each on an Illumina NextSeq instrument, which produced paired 75-base pair (bp) reads; that is, 150 bases per DNA fragment. The read pairs were analyzed with two different metagenomics classification engines, Kraken^5^ and Centrifuge,^6^ and visualized using the Pavian software tool^7^ (Table 2; Supplementary Fig. S1). The reads from each group were analyzed by a bioinformatics team (FPB, JL, SLS) who were masked with respect to pathology and microbiology diagnostic data until after reporting the initial Kraken calls shown in Table 1.

**Table 2:**
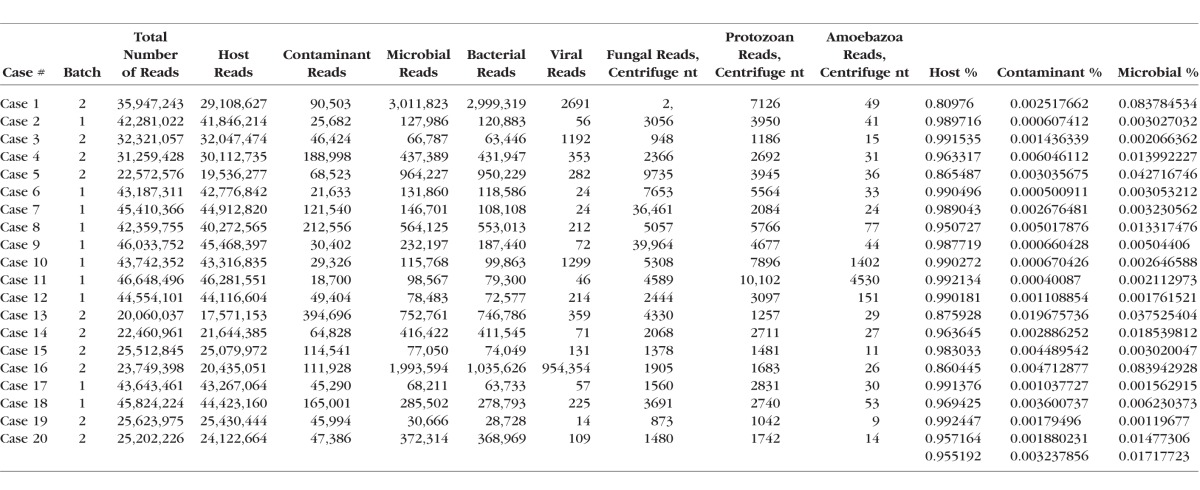
Summary of Sequence Read Numbers

The Kraken database contained the human genome (version GRCh38), mouse genome (GRCm38), 4111 bacterial genomes (representing 1818 distinct species), 5412 viral reference genomes plus 84,272 viral strain genomes, 202 archaeal genomes, and 25 selected genomes of eukaryotic pathogens. To avoid false-positives caused by matches to low-complexity sequences, we masked all genomes using the National Center for Biotechnology Information (NCBI) DustMasker program, which is part of the BLAST package. Furthermore, we added 5426 sequences from the NCBI UniVec database of artificial vectors, adapters, linkers, and primers, and 3652 sequences from the EmVec vector database. In total, the Kraken database was based on 24 billion base pairs (Gbp) of sequences compiled into a 154 gigabyte (GB) index. The computational analysts were blinded to pathology and other clinical data until the initial analysis was complete.

The Centrifuge database was based on NCBI's nonredundant nucleotide collection (nt) downloaded on February 28, 2016. This database contains finished genomes as well as draft genomes and partial sequences. In total, it comprises nearly one million species across 75 thousand genera, including many eukaryotes. The 110 Gbp of sequence data were compiled into a 69 GB Centrifuge index. Centrifuge was run with the setting “k = 1” to return the lowest common ancestor of the hits for each sequence. All sequence mappings with a minimum alignment length of 50 bp were summarized to a Kraken-like report with “centrifuge-kreport.”

During the course of the study, we built a second database for Kraken containing 255 genomes of eukaryotic pathogens from EuPathDB,^8^ most of which were not present in Kraken's bacterial/viral database. For the second group of 10 samples, Kraken was run using both databases, and the results were analyzed jointly to identify candidate pathogens.

The results of Kraken and Centrifuge were analyzed and visualized with the Pavian software tool, which allows cross-sample comparison and normalization. To rank the microbial identifications, we first removed all taxa that matched to any chordate (taxonomy ID 7111 and below) or artificial sequences (taxonomy ID 81077 and below). We then calculated the relative abundance \newcommand{\bialpha}{\boldsymbol{\alpha}}\newcommand{\bibeta}{\boldsymbol{\beta}}\newcommand{\bigamma}{\boldsymbol{\gamma}}\newcommand{\bidelta}{\boldsymbol{\delta}}\newcommand{\bivarepsilon}{\boldsymbol{\varepsilon}}\newcommand{\bizeta}{\boldsymbol{\zeta}}\newcommand{\bieta}{\boldsymbol{\eta}}\newcommand{\bitheta}{\boldsymbol{\theta}}\newcommand{\biiota}{\boldsymbol{\iota}}\newcommand{\bikappa}{\boldsymbol{\kappa}}\newcommand{\bilambda}{\boldsymbol{\lambda}}\newcommand{\bimu}{\boldsymbol{\mu}}\newcommand{\binu}{\boldsymbol{\nu}}\newcommand{\bixi}{\boldsymbol{\xi}}\newcommand{\biomicron}{\boldsymbol{\micron}}\newcommand{\bipi}{\boldsymbol{\pi}}\newcommand{\birho}{\boldsymbol{\rho}}\newcommand{\bisigma}{\boldsymbol{\sigma}}\newcommand{\bitau}{\boldsymbol{\tau}}\newcommand{\biupsilon}{\boldsymbol{\upsilon}}\newcommand{\biphi}{\boldsymbol{\phi}}\newcommand{\bichi}{\boldsymbol{\chi}}\newcommand{\bipsi}{\boldsymbol{\psi}}\newcommand{\biomega}{\boldsymbol{\omega}}\({\mathop x\limits^\sim _{ij}}\) of species \newcommand{\bialpha}{\boldsymbol{\alpha}}\newcommand{\bibeta}{\boldsymbol{\beta}}\newcommand{\bigamma}{\boldsymbol{\gamma}}\newcommand{\bidelta}{\boldsymbol{\delta}}\newcommand{\bivarepsilon}{\boldsymbol{\varepsilon}}\newcommand{\bizeta}{\boldsymbol{\zeta}}\newcommand{\bieta}{\boldsymbol{\eta}}\newcommand{\bitheta}{\boldsymbol{\theta}}\newcommand{\biiota}{\boldsymbol{\iota}}\newcommand{\bikappa}{\boldsymbol{\kappa}}\newcommand{\bilambda}{\boldsymbol{\lambda}}\newcommand{\bimu}{\boldsymbol{\mu}}\newcommand{\binu}{\boldsymbol{\nu}}\newcommand{\bixi}{\boldsymbol{\xi}}\newcommand{\biomicron}{\boldsymbol{\micron}}\newcommand{\bipi}{\boldsymbol{\pi}}\newcommand{\birho}{\boldsymbol{\rho}}\newcommand{\bisigma}{\boldsymbol{\sigma}}\newcommand{\bitau}{\boldsymbol{\tau}}\newcommand{\biupsilon}{\boldsymbol{\upsilon}}\newcommand{\biphi}{\boldsymbol{\phi}}\newcommand{\bichi}{\boldsymbol{\chi}}\newcommand{\bipsi}{\boldsymbol{\psi}}\newcommand{\biomega}{\boldsymbol{\omega}}\(i\) in sample \newcommand{\bialpha}{\boldsymbol{\alpha}}\newcommand{\bibeta}{\boldsymbol{\beta}}\newcommand{\bigamma}{\boldsymbol{\gamma}}\newcommand{\bidelta}{\boldsymbol{\delta}}\newcommand{\bivarepsilon}{\boldsymbol{\varepsilon}}\newcommand{\bizeta}{\boldsymbol{\zeta}}\newcommand{\bieta}{\boldsymbol{\eta}}\newcommand{\bitheta}{\boldsymbol{\theta}}\newcommand{\biiota}{\boldsymbol{\iota}}\newcommand{\bikappa}{\boldsymbol{\kappa}}\newcommand{\bilambda}{\boldsymbol{\lambda}}\newcommand{\bimu}{\boldsymbol{\mu}}\newcommand{\binu}{\boldsymbol{\nu}}\newcommand{\bixi}{\boldsymbol{\xi}}\newcommand{\biomicron}{\boldsymbol{\micron}}\newcommand{\bipi}{\boldsymbol{\pi}}\newcommand{\birho}{\boldsymbol{\rho}}\newcommand{\bisigma}{\boldsymbol{\sigma}}\newcommand{\bitau}{\boldsymbol{\tau}}\newcommand{\biupsilon}{\boldsymbol{\upsilon}}\newcommand{\biphi}{\boldsymbol{\phi}}\newcommand{\bichi}{\boldsymbol{\chi}}\newcommand{\bipsi}{\boldsymbol{\psi}}\newcommand{\biomega}{\boldsymbol{\omega}}\(j\) by dividing the read counts (times 100) by the total number of microbial species reads in that sample. A robust z-score was calculated from the relative abundances using the following formula:

\newcommand{\bialpha}{\boldsymbol{\alpha}}\newcommand{\bibeta}{\boldsymbol{\beta}}\newcommand{\bigamma}{\boldsymbol{\gamma}}\newcommand{\bidelta}{\boldsymbol{\delta}}\newcommand{\bivarepsilon}{\boldsymbol{\varepsilon}}\newcommand{\bizeta}{\boldsymbol{\zeta}}\newcommand{\bieta}{\boldsymbol{\eta}}\newcommand{\bitheta}{\boldsymbol{\theta}}\newcommand{\biiota}{\boldsymbol{\iota}}\newcommand{\bikappa}{\boldsymbol{\kappa}}\newcommand{\bilambda}{\boldsymbol{\lambda}}\newcommand{\bimu}{\boldsymbol{\mu}}\newcommand{\binu}{\boldsymbol{\nu}}\newcommand{\bixi}{\boldsymbol{\xi}}\newcommand{\biomicron}{\boldsymbol{\micron}}\newcommand{\bipi}{\boldsymbol{\pi}}\newcommand{\birho}{\boldsymbol{\rho}}\newcommand{\bisigma}{\boldsymbol{\sigma}}\newcommand{\bitau}{\boldsymbol{\tau}}\newcommand{\biupsilon}{\boldsymbol{\upsilon}}\newcommand{\biphi}{\boldsymbol{\phi}}\newcommand{\bichi}{\boldsymbol{\chi}}\newcommand{\bipsi}{\boldsymbol{\psi}}\newcommand{\biomega}{\boldsymbol{\omega}}\[{z_{ij}} = {{{{\mathop x\limits^\sim }_{ij}} - {\rm{median}}({{\mathop x\limits^\sim }_{i.}})} \over {{\rm{max}}({\rm{mad}}\left( {{{\mathop x\limits^\sim }_{i.}}} \right),0.001)}}{\rm {,}}\]

where \newcommand{\bialpha}{\boldsymbol{\alpha}}\newcommand{\bibeta}{\boldsymbol{\beta}}\newcommand{\bigamma}{\boldsymbol{\gamma}}\newcommand{\bidelta}{\boldsymbol{\delta}}\newcommand{\bivarepsilon}{\boldsymbol{\varepsilon}}\newcommand{\bizeta}{\boldsymbol{\zeta}}\newcommand{\bieta}{\boldsymbol{\eta}}\newcommand{\bitheta}{\boldsymbol{\theta}}\newcommand{\biiota}{\boldsymbol{\iota}}\newcommand{\bikappa}{\boldsymbol{\kappa}}\newcommand{\bilambda}{\boldsymbol{\lambda}}\newcommand{\bimu}{\boldsymbol{\mu}}\newcommand{\binu}{\boldsymbol{\nu}}\newcommand{\bixi}{\boldsymbol{\xi}}\newcommand{\biomicron}{\boldsymbol{\micron}}\newcommand{\bipi}{\boldsymbol{\pi}}\newcommand{\birho}{\boldsymbol{\rho}}\newcommand{\bisigma}{\boldsymbol{\sigma}}\newcommand{\bitau}{\boldsymbol{\tau}}\newcommand{\biupsilon}{\boldsymbol{\upsilon}}\newcommand{\biphi}{\boldsymbol{\phi}}\newcommand{\bichi}{\boldsymbol{\chi}}\newcommand{\bipsi}{\boldsymbol{\psi}}\newcommand{\biomega}{\boldsymbol{\omega}}\({\mathop x\limits^\sim _{i.}}\) are the relative abundances for species \newcommand{\bialpha}{\boldsymbol{\alpha}}\newcommand{\bibeta}{\boldsymbol{\beta}}\newcommand{\bigamma}{\boldsymbol{\gamma}}\newcommand{\bidelta}{\boldsymbol{\delta}}\newcommand{\bivarepsilon}{\boldsymbol{\varepsilon}}\newcommand{\bizeta}{\boldsymbol{\zeta}}\newcommand{\bieta}{\boldsymbol{\eta}}\newcommand{\bitheta}{\boldsymbol{\theta}}\newcommand{\biiota}{\boldsymbol{\iota}}\newcommand{\bikappa}{\boldsymbol{\kappa}}\newcommand{\bilambda}{\boldsymbol{\lambda}}\newcommand{\bimu}{\boldsymbol{\mu}}\newcommand{\binu}{\boldsymbol{\nu}}\newcommand{\bixi}{\boldsymbol{\xi}}\newcommand{\biomicron}{\boldsymbol{\micron}}\newcommand{\bipi}{\boldsymbol{\pi}}\newcommand{\birho}{\boldsymbol{\rho}}\newcommand{\bisigma}{\boldsymbol{\sigma}}\newcommand{\bitau}{\boldsymbol{\tau}}\newcommand{\biupsilon}{\boldsymbol{\upsilon}}\newcommand{\biphi}{\boldsymbol{\phi}}\newcommand{\bichi}{\boldsymbol{\chi}}\newcommand{\bipsi}{\boldsymbol{\psi}}\newcommand{\biomega}{\boldsymbol{\omega}}\(i\) in all samples, and \newcommand{\bialpha}{\boldsymbol{\alpha}}\newcommand{\bibeta}{\boldsymbol{\beta}}\newcommand{\bigamma}{\boldsymbol{\gamma}}\newcommand{\bidelta}{\boldsymbol{\delta}}\newcommand{\bivarepsilon}{\boldsymbol{\varepsilon}}\newcommand{\bizeta}{\boldsymbol{\zeta}}\newcommand{\bieta}{\boldsymbol{\eta}}\newcommand{\bitheta}{\boldsymbol{\theta}}\newcommand{\biiota}{\boldsymbol{\iota}}\newcommand{\bikappa}{\boldsymbol{\kappa}}\newcommand{\bilambda}{\boldsymbol{\lambda}}\newcommand{\bimu}{\boldsymbol{\mu}}\newcommand{\binu}{\boldsymbol{\nu}}\newcommand{\bixi}{\boldsymbol{\xi}}\newcommand{\biomicron}{\boldsymbol{\micron}}\newcommand{\bipi}{\boldsymbol{\pi}}\newcommand{\birho}{\boldsymbol{\rho}}\newcommand{\bisigma}{\boldsymbol{\sigma}}\newcommand{\bitau}{\boldsymbol{\tau}}\newcommand{\biupsilon}{\boldsymbol{\upsilon}}\newcommand{\biphi}{\boldsymbol{\phi}}\newcommand{\bichi}{\boldsymbol{\chi}}\newcommand{\bipsi}{\boldsymbol{\psi}}\newcommand{\biomega}{\boldsymbol{\omega}}\({\rm{mad}}\left( {{{\mathop {x}\limits^\sim }_{i.}}} \right)\) is the median absolute deviation, a robust measure of the variability. The minimum value of 0.001 for the divisor was chosen to avoid division by zero (when species are not present in most samples), and the inflation of scores when the variability of the relative abundance is very low. The minimum value of 0.001 was found to give the best ranking for this sample set (data not shown). The species then were ranked according to the z-score.

The intuition behind the z-score is that we expect to see true pathogens only in a subset of the samples, while contaminants or DNA from surface microbiome organisms tend to occur in many more samples. A high z-score indicates that more reads than expected were seen in a particular sample, which usually corresponds to a true signal. When searching against the nt database, hundreds to thousands of taxons might be hit erroneously (due to low complexity sequences or to contaminants in the database), and comparing across samples provides greater power to see the true pathogen above the background of contamination and/or commensal organisms. Supplementary Table S2 shows the z-scores for the top species with Centrifuge in this study, and Supplementary Table S3 shows the raw read counts for those same species. We additionally tested the stability of the z-score ranking using bootstrap selection of subsets of the samples (Supplementary Fig. S2).

In every case, we realigned selected reads using BLAST, a slow but very sensitive method, against the full NCBI nucleotide database to validate our initial diagnoses. The reads chosen were simply the first 20 identified by Kraken as belonging to the suspected pathogen. In a few cases, these BLAST searches detected a closely related genome that was missing from the Kraken database, allowing us to realign the sequences to a particular genome and change the initial diagnosis. For case 9, the reads that did not match human or contaminant sequences were aligned with bowtie2^9^ against the *Aspergillus flavus* strain NRRL3557 (accession code GCF_000006275.2, total sequence length 36,892,344 bp), and the *A. oryzae* strain RIB40 (GCA_000184455.3, total sequence length 37,912,014 bp). In cases 3 and 4, nonhuman/noncontaminant reads were aligned to *Mycobacterium abscessus* strain ATCC 19977 (GCF_000069185.1, total sequence length 5,090,491 bp) and *M. chelonae* strain CCUG 47445 (GCF_001632805.1, total sequence length 5,029,817 bp). In case 3, of the 194,482 read pairs, 1118 and 8468 reads mapped to *M. abscessus* and *M. chelonae*, respectively. In case 4, of the 637,117 read pairs, 5407 and 48,888 reads mapped. In case 6, the nonhuman/noncontaminant reads were aligned with bowtie2 to *Nectria haematococca/Fusarium solani* strain mpVI 77-13-4 (GCF_000151355.1, total sequence lengths 51,286,497 bp).

### Confirmatory Testing by Quantitative PCR (qPCR)

DNA was extracted from paraffin sections as described above. Previously published primer sequences were used to detect *Staphylococcus aureus* (Forward: 5′-TCGGTACACGATATTCTTCAC-3′, Reverse: 5′-ACTCTCGTATGACCAGCTTC-3′) and cytomegalovirus (Forward: 5′-GCGGTGGTTGCCCAACAGGA-3′, Reverse: 5′-ACGACCCGTGGTCATCTTTA-3′).^10,11^ Reactions were performed in triplicate with β-Actin as a control in a iQ5 Multicolor real-time PCR detection system (Bio-Rad, Hercules, CA, USA), using SYBR Green (Applied Biosystems, Foster City, CA, USA) as fluorescent dye.

## Results

We chose to analyze corneal infections by a range of organisms, grouped below into several categories. For each group, we highlight in greater detail the clinical, pathologic, and sequence data from one or two cases. Overall clinical, pathologic, and NGS data are summarized in Table 1, while Table 2 provides an overview of the number of reads across the samples and categories, and Supplementary Table S1 the Kraken read numbers for species named in Table 1. On average, 96% of the NGS reads were classified as human (range, 81%–99%), 0.3% corresponded to known contaminant sequences in the database, 1.7% (range, 0.1%–8%) represented microbial sequences, and the remainder could not be classified (Table 2). Note that in the two cases with 8% nonhost reads (cases 1 and 16), the vast majority were from the pathogen causing the infection.

### Bacterial and Mycobacterial Keratitis

**Figure 1:**
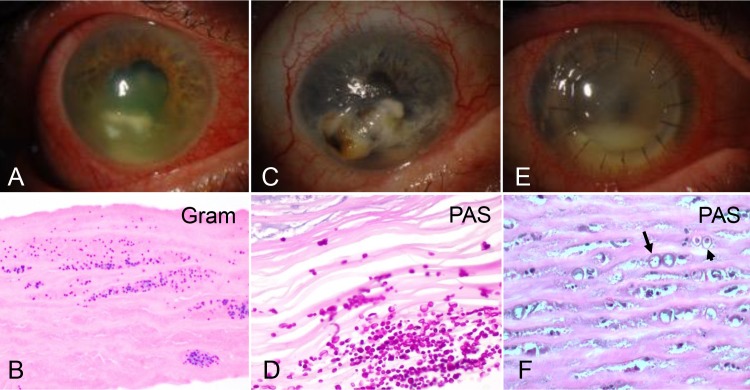
Clinical and microscopic appearance of selected cases. (A) In case 1, the patient's cornea showed inflammation and ulceration. (B) Microscopic examination of case 1 revealed Gram-positive cocci in the stroma (original magnification, ×400). (C) Severe corneal ulceration in case 7 ultimately led to endophthalmitis and enucleation. (D) Periodic acid-Schiff (PAS) stains highlight numerous yeast forms in the corneal stroma (original magnification, ×400). (E) In case 12, 1 month after an initial penetrating keratoplasty, worsening graft edema and haze was accompanied by a hypopyon. (F) Numerous Acanthamoeba cysts were present, some of which were empty (arrow), while others contained organisms (arrowhead, original magnification ×400).

Our study included two cases of keratitis caused by Gram-positive cocci (Table 1). Representative clinical and microscopic images of the cornea in case 1 are shown in Figures 1A and 1B, respectively. This patient had cornea ulceration as well as a clinical history of herpetic infection and was being treated with oral acyclovir, fortified vancomycin, and tobramycin at the time of penetrating keratoplasty, although no direct testing for virus had been performed. No organisms grew in culture, but the specimen sent to the microbiology laboratory and the corneal button examined in ophthalmic pathology contained gram-positive cocci visualized microscopically. Our initial NGS assay identified *S. aureus* (1,795,339 reads) and human herpesvirus 1 (HSV-1, 692 reads), as well as a lower count of HSV-5 (cytomegalovirus) sequences (330 reads), which were not regarded as clearly significant on the initial analysis. The second case, in which group B Streptococcus was grown from a vitreous culture, showed *S. agalactiae* on NGS analysis (5782 reads).

We also examined two cases of atypical mycobacterial keratitis. In case 3, standard aerobic and anaerobic cultures were negative, and mycobacterial cultures were not performed, but acid-fast bacilli were detected microscopically on Fite stains in the surgical material. *M. abscessus* (12,070 reads) and HSV-5 (1099 reads) were found by NGS. In case 4, which clinically involved the conjunctiva and cornea, heavy *M. chelonae* was identified on culture. In this case only a small limbal biopsy approximately 1 mm in greatest dimension was taken, with acid-fast organisms found microscopically. Kraken initially identified *M. abscessus* in the NGS sequences (2158 reads), along with HSV-1 (285 reads). Realignment of the sequences to a larger database (see Methods) revealed that *M. chelonae* was a better match, and, therefore, we reported *M. chelonae* as our final NGS diagnosis before unmasking the clinical diagnosis. Importantly, even in this small biopsy the total number of sequence reads (31.3 million) as well as the number of mycobacterial sequences (4120 total mycobacterial and 2158 *M. abcessus* reads) was relatively high (Supplementary Table S1). Thus, the concordance between NGS and clinical diagnostics for these bacterial and mycobacterial keratitis cases was 100%.

### Fungal Keratitis

Five cases of fungal keratitis were examined (Table 1). In case 5, corneal tissue around a failed keratoprosthesis was submitted, and yeast forms and gram-positive cocci were identified microscopically, although only a single small focus of bacteria was noted in the multiple fragments of adherent tissue. The initial NGS results from Kraken were inconclusive, but secondary analysis using an expanded eukaryotic pathogen database (see Methods) found *Candida parapsilosis*, a species that was not in the initial Kraken database. The absence of significant bacterial sequences may reflect their focal involvement of the specimen. In case 6 *F. solani* was cultured and fungal forms seen microscopically. *F. solani* was not identified in the initial NGS analysis because no Fusarium genome was present in the database used. Case 7 was a globe enucleated due to a corneal ulcer which ultimately perforated (Fig. 1C), with *C. albicans* identified by culture and NGS (46,860 reads), and yeast forms also confirmed in the cornea microscopically (Fig. 1D). In case 8, *Curvularia clavata* was cultured and fungal forms detected microscopically. No fungal DNA sequences were identified by NGS, but *C. clavata* has never been sequenced, therefore it cannot yet be detected by an NGS method. On the initial Kraken analysis, *M. smegmatis*, a rapidly growing acid-fast organism of low virulence, was highlighted.^12^ However, similar or greater numbers of sequence reads from this organism were identified in many samples, suggesting it is less likely to be pathogenic, and may have contaminated the samples at some point during processing (Supplementary Table S2). Finally, in case 9, fungal forms were seen microscopically and *Aspergillus* was detected by culture and NGS. *A. flavus* was found on culture, while NGS identified *A. fumigatus* (469 reads), which was the closest relative to *A. flavus* in the Kraken database and belongs to the same taxonomic group.^13^

We also examined a cornea (case 10) infected with microsporidia, eukaryotic parasitic organisms previously thought to be protozoa but now believed to be related more closely to fungi.^14^ This tissue had been sent previously for analysis at the Center for Disease Control (CDC), where direct immunofluorescence had been used to establish a diagnosis of *Anncaliia algerae*. On secondary analysis by Kraken with the expanded eukaryotic pathogen database, this case showed an overwhelmingly strong signal (155,152 reads) of *A. algerae*. Thus, Kraken analyses using the expanded database was concordant with the clinical diagnosis in four of six cases (66%), with the two failures representing fungi absent from database.

### Acanthamoeba

Three cases of *Acanthamoeba* were examined, including one in which two keratoplasties and extensive medical therapy was required before the organisms ultimately were cleared (Fig. 1E). All contained cysts or organisms on microscopic examination (Fig. 1F), and in two of these *Acanthamoeba* also were identified by culture in the microbiology laboratory, although in case 12 only early cultures were positive while those at surgery were negative. The Kraken database used for initial NGS analysis did not include *Acanthamoeba* sequences, and the main organisms identified were vaccinia virus and *Mycoplasma fermentans* (case 11), a bacterial endosymbiont of *Acanthamoeba* known as UWC8 (case 12), and no definitive infectious organisms in case 13.

Because of the detection of the endosymbiont in case 12, the initial analysis pointed to *Acanthamoeba* despite the absence of its genome from the database. With the database with eukaryotic pathogens, Kraken identified 8252, 27,860, and 967 reads at the *Acanthamoeba* genus level, with 509, 1505, and 53 specific to the *A. castellanii* species, in cases 11, 12, and 13 respectively, resulting in 100% concordance with clinical diagnosis.

### Viral Keratitis and Controls

Three cases with clinical and/or microscopic features of herpetic keratitis were examined. In two of these (cases 14 and 16) HSV-1 had been detected by culture at some point during the clinical course. Case 15 had a clinical history of herpes zoster ophthalmicus and presumed herpetic keratitis, but only bacterial and fungal cultures had been performed and were negative; the cornea was scarred in a nonspecific fashion. Only case 16 showed significant numbers of HSV-1 sequences on initial NGS analysis (947,786 reads, Supplementary Table S1), for a concordance rate of only 33% with clinical diagnosis.

### Controls

We also sequenced DNA extracted from four penetrating keratoplasty specimens with no clinical or microscopic suspicion of infection (cases 17–20). Two of these showed rare sequences suggesting possible pathogenic involvement by *M. smegmatis* (case 18, 223 reads) and *S. aureus* (case 20, 1869 reads). These also could represent organisms of the endogenous surface microbiome, or contaminants from the skin or other sources. To address the possibility that environmental organisms or DNA were contaminating the samples during specimen grossing, fixation, or processing, we examined three corneas that were divided in a sterile fashion immediately postoperatively, with one portion frozen and the other processed routinely for microscopic diagnosis. As shown in Supplementary Table S4, modest differences were seen between the sterile and formalin-fixed, paraffin-embedded specimens, but the number of pathogen reads did not approach those seen in truly infected specimens using either processing method

### Reanalysis With Centrifuge

**Figure 2:**
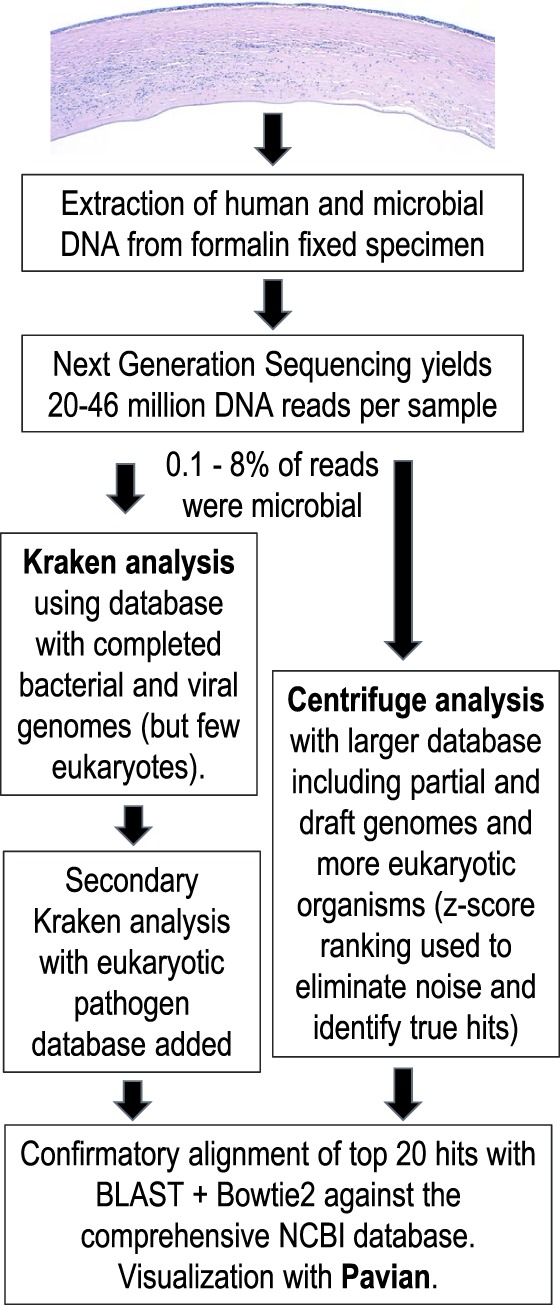
Strategy for sequencing and computational analysis.

After the initial results, generated while the computational analysis team was masked with respect to clinical diagnosis, it was decided to use another strategy and sequences from a broader spectrum of organisms (see Table 1, column “NGS Reanalysis Results,” and Supplementary Tables S2, S3). The recently-developed Centrifuge metagenomics classifier^6^ enables queries against much larger sequence sets—assuming a fixed amount of computer memory—than Kraken (Fig. 2). We compiled an index from the NCBI's comprehensive nucleotide database, which contains sequences from hundreds of eukaryotic pathogens that were missing from the Kraken database used in the initial analysis. We calculated a robust z-score for each identification across the samples (see Methods), and extracted the 25 species with the highest z-score (see Supplementary Table S2 for the z-scores across the samples, and Supplementary Table S3 for NGS read numbers for the species from S2). This additional statistical normalization procedure is necessary because the nt database contains much more noise caused by matches to off-target sequences. For example, the Centrifuge analysis identified matches to human HSV1 in all 20 samples, but manual inspection revealed that most of these were false-positives, and none was ranked highly by z-scores. The z-score ranking tends to eliminate noise and put true positive classifications at the top of the ranking.

Using this approach, we found strong signals for a number of eukaryotic pathogens that we did not detect in our initial NGS assay in the first round, including *Acanthamoeba* in cases 10 to 12, *C. parapsilosis* in case 5, and *F. solani* in case 6. Note that the nt database contains sequences for *F. solani* labelled as both *N. haematococca* (taxonomy ID 140110) and *F. solani* (taxonomy ID 169388), both members of the *F. solani* species complex. *N. haematococca* is the teleomorph of *F. solani*, and genomically identical. For the purpose of identification, we can sum their reads. A targeted alignment of the nonhuman reads from case 6 to the *F. solani* genome identified 8341 reads from that organism. Interestingly, in case 9 *A. oryzae* was assigned more reads than *A. flavus*, probably due to missing sequences from the draft genome of *A. flavus* that are present in the *A. oryzae* draft genome. Indeed, the *A. oryzae* genome has over three times more sequence data in the nt database than *A. flavus*. When we independently aligned the reads against two assemblies of *A. oryzae* and *A. flavus* with similar total sequence lengths, the number of mapped reads was nearly identical and comprised over 9% of all nonhuman/noncontaminant reads in this sample. Finally, we detected a fungus in case 8, where the signal was strongest for Candida/*Clavispora lusitaniae* (638 reads). As mentioned above, *C. clavata*, which was detected in the microbiology laboratory culture, has not had its genome sequenced and, thus, cannot be detected by NGS at this time. In case 4, where the initial results suggested *M. abscessus*, the diagnosis was revised to *M. chelonae* during the validation step for the initial results, as explained above. A targeted alignment of the nonhuman reads to genomes of both species confirmed that eight to nine times more reads align to *M. chelonae* in cases 3 and 4 (Supplementary Table S3).

In the other infected cases, the identifications from the first round were confirmed; thus, 14 of 16 cases (88%) had Centrifuge results concordant with clinical diagnosis. In cases 17 to 20, which were specimens from failed grafts, modest numbers of reads from a range of organisms were identified, as shown in Supplementary Table S3.

Because in some cases NGS identified sequences from a pathogen not detected previously with conventional methods, confirmatory testing was performed for two of the potential false-positives, *S. aureus* and CMV, in eight cases using quantitative PCR. In case 1, gram positive cocci had been identified in the microbiology and surgical pathology specimens, corresponding to the *S. aureus* identified by Kraken and Centrifuge analyses. *S. aureus* also was identified as a possible pathogen in the Centrifuge analysis of case 20, although Kraken analysis of that case did not clearly support a diagnosis of *S. aureus*. Our qPCR analysis confirmed the presence of *S. aureus* DNA in case 1, but did not detect it in case 20 or any other case examined (Supplementary Fig. S1). CMV was detected by Kraken and Centrifuge in case 3, but had not been tested for clinically, and also was confirmed by qPCR. Case 1, however, in which Centrifuge but not Kraken had resulted in a positive call for CMV, did not show this virus by qPCR (Supplementary Fig. S3).

## Discussion

In this retrospective study, we analyzed 16 corneas infected with a range of pathogens to investigate the ability of high throughput sequencing coupled with advanced computational analysis to identify multiple types of organisms in a single test. Our initial NGS analysis using Kraken was able to detect sequences from organisms largely corresponding to those found clinically in four of four bacterial/mycobacterial cases, two of six fungal cases, and one of three herpetic keratitis cases. In this initial analysis, the absence of adequate sequences from some species in the Kraken reference database resulted in imperfect matches.

The paucity of eukaryotic sequences in the initial Kraken database proved problematic, but this was addressed easily by building a second database using a collection of eukaryotic pathogens. No *Acanthamoeba* sequences were matched initially due this lack of database coverage, but an endosymbiont of *Acanthamoeba* known as UWC8 was present. *Acanthamoeba* sequences then were added to the Kraken database, and the pathogen was identified in all three infected samples. The Centrifuge metagenomics classifier, which queries a broader set of sequences, was used in a second round of analyses and detected the eukaryotic pathogens in all the samples that were inconclusive in the first round, including *Acanthamoeba* in these three cases; *M. chelonae* in cases 3 and 4; Fusarium and Candida in the fungal keratitis cases 5, 6, and 8; and the microsporidia *A. algerae* in case 10. The correct organisms also were identified in cases 5 and 10 by Kraken in a secondary analysis including an expanded eukaryotic database.

Although Centrifuge's larger database allowed it to be more sensitive on initial analysis, the large number of draft genomes, many with contaminating sequences, result in a higher false-positive rate than Kraken. For example, moderate numbers of HSV-1 reads were identified in all samples, but manual alignment revealed that most of these were false-positives representing low-complexity matches clustered in a few locations of the HSV-1 genome. The z-score ranking also can be used to exclude false-positive matches when a sufficient number of similar samples is available for comparison. Centrifuge (but not Kraken) also detected sufficient numbers of DNA reads for CMV in case 1 and *S. aureus* in case 20 to suggest infection, but organismal DNA was not confirmed using qPCR. Thus thresholds for Centrifuge calls will need to be adjusted to prevent false-positives after additional cases are analyzed.

Our findings nevertheless support the general feasibility of using metagenomic sequencing plus computational analysis to identify DNA sequences from a wide range of infectious organism in the cornea and other ocular tissues. In addition to facilitating basic research on infectious keratitis, NGS eventually could become a standard diagnostic assay in clinical microbiology laboratories. However, this approach is dependent on sequences from the pathogens, or closely-related organisms, being present in the reference databases queried. While sequences for thousands of infectious agents already are available, and the reference databases needed for such studies are expanding, additional efforts in this area will be required for metagenomics efforts to reach their broadest potential.^15,16^

Thousands of pathogen genomes have been sequenced, but most of the genomes have not yet been assembled fully, especially the large genomes of eukaryotic pathogens.^17^ Partial genome assemblies, which have their sequence data in many pieces, often include contaminating foreign sequences. Mapping sequencing results against a metagenomics database with only complete genomes, as we did in the initial analysis with Kraken, thus often gives clearer results, but limits the range of detectable species. Even if there is a detection, the researcher must consider whether the signal could be off-target. Classifiers assign reads to the closest genome in the database, and if an organism in a tissue sample represents a species that does not have a genome in the database, reads will be assigned to sufficiently close species, as was seen with the initial *M. abscessus* assignments in our case 3.

Novel metagenomics classifiers, such as Centrifuge, enable efficient search against much larger sequence sets.^6^ While there is a much better chance that the database then includes the sequence of the target pathogen, the results usually are messier due to contamination in the database and off-target hits from the classifier. We demonstrated that by using a statistical filter to extract the outliers in the sea of results, common contaminants can be dismissed more easily and the true signal can become clear. Visualization tools, such as Pavian, which includes the statistical filtering method used in our study, can be a great help in this regard, and an illustration of output from this program is shown in Supplementary Figure S1.^7^ Another metagenomics classifier, known as SMART, which also can index the entire GenBank database, recently was reported and used to characterize the distribution of organisms using sequences from a human microbiome project conjunctival sample.^18^

An alternative approach for metagenomics classification first assembles short NGS reads into larger contiguous sequences (contigs).^19^ Organisms must be present abundantly in a sample before this assembly approach will yield longer contiguous sequences. The largest and most-covered contigs then can be queried against a database. Since the assembly process reduces the sequence set dramatically, very sensitive but slower methods, such as BLAST, can be used. While assembly-based metagenomics classification might be more sensitive in detecting abundant organisms that are not in the database, they give few results when the genome of interest is covered sparsely, which almost always will be the case for the low volumes of DNA available from biopsy specimens.

A range of practical issues will need to be addressed before NGS assays can be used as routine diagnostics for corneal infection. For each organism, thresholds for the number of DNA sequences corresponding to significant infectious loads in the cornea will need to be established. True contamination and the levels of commensal organisms of the ocular surface also will need to be better understood. For example, we identified contamination by rat or mouse sequences in case 7, and by citrus lemon in case 17. Very low levels of *Acanthamoeba* sequence reads also were identified in several specimens run in the same batch as cases 11 to 13, but not in runs of the second batch, suggesting that some cross-contamination can occur when groups of specimens are processed together.

It also will be necessary to define the endogenous corneal microbiota, which varies between individuals. In our four control cases, we detected significant numbers of sequences from a range of organisms, including *Pencillium digitatum*, *Bifidobacterium scarvodii*, *Cornyebacterium imitans*, *M. smegmatis* and *S. aureus*, although the latter was not confirmed when tested for using qPCR. Infectious keratitis and other noninfectious causes of corneal inflammation and scarring also likely will alter the composition of organisms on the corneal surface; thus, it will take some time to form a fuller picture of the corneal microbiome. A number of studies using 16S ribosome sequencing or other techniques have begun to define the ocular surface microbiome, but it remains poorly understood in health and disease.^20–22^

Studies using cell culture have reported Gram-positive species, such as *Propionibacterium, Corynebacterium, Staphylococcus*, and *Streptococcus* on the surface of the eye, although some have suggested that fungi also can be present.^23,24^ Dong et al.^25^ used deep sequencing of amplified 16S rRNA gene libraries from surface swab DNA to identify bacteria in healthy human conjunctiva, and reported *Pseudomonas*, *Bradyrhizobium*, *Propionibacterium, Acinetobacter*, and *Corynebacterium* as the most common species identified. Doan et al.^22^ used bacterial culture, 16S rRNA sequencing and another sequencing technique, known as biome representational in silico karyotyping (BRiSK), to analyze conjunctival swabs from 107 healthy volunteers, with *Corynebacterium*, *Propionibacterium*, and coagulase-negative *Staphylococci* reported as the predominant organisms. We identified generally low numbers of reads corresponding to several of these organisms, including *Staphylococcus*, *Streptococcus*, and *Corynebacterium*, in many of our control and infected corneal specimens.

In addition to serving as a diagnostic tool, this technique ultimately will make it possible to study interactions between pathogenic and nonpathogenic infections organisms, or between multiple pathogens. Indeed, in some corneas being treated for nonviral pathogens, we identified viral sequences that may have had a role in clinical findings. In case 3, CMV was found in addition to mycobacterial keratitis and confirmed by qPCR. Interrogation of additional cases should provide a broader picture of what viral, bacterial, and eukaryotic organisms often are found together in distinct clinical contexts.

In summary, these preliminary studies establish the feasibility of using metagenomic NGS of DNA extracted from routine formalin-fixed clinical specimens to identify bacteria, fungi, amoeba, and viruses associated with pathogenic corneal infections. This method holds great promise for the relatively rapid detection of organisms, including rare pathogens and cases in which cultures were not attempted or failed to yield positive results. Additional prospective studies with more detailed confirmatory testing will be required, however, particularly as some organisms we detected by NGS but not identified clinically could not be confirmed by qPCR, including CMV identified by Centrifuge in case 1 and *S. aureus* in case 20. While most of the specimens examined in our report represented full corneal buttons, the one superficial biopsy examined yielded similar numbers of sequence reads, indicating that the method can work even in very small specimens. Indeed, the greatest use of this approach may involve its use with fresh tissue scrapings taken from the patient's cornea, as opposed to surgically acquired biopsies or keratoplasty specimens. The potential to diagnose infections in small biopsies using NGS was highlighted in two recent studies using small intraocular fluid samples. One group identified torque teno virus in a number of culture-negative endophthalmitis cases.^26^ The second reported a case of chronic idiopathic bilateral uveitis in which rubella was identified using metagenomics deep sequencing and subsequently confirmed using other methods.^27^

## Supplementary Material

Supplement 1

Supplement 2

Supplement 3

Supplement 4
