# Supplementary material for: Identifying Corneal Infections in Formalin-Fixed Specimens Using Next Generation Sequencing
Source: Invest Ophthalmol Vis Sci. 2018 Jan;59(1):280–8. doi: 10.1167/iovs.17-21617 (PMC5770184; doi:10.1167/iovs.17-21617)

Pavian

127.0.0.1:3753

Florian

Pavian metagenomics data explorer

Select sample set

cornea-samples/centrifuge

Search microbes ...

- Data Input
- Results Overview
- Comparison
  - All data
  - Bacteria and Archaea
  - Viruses
  - Eukaryotes
  - Eukaryotes/Fungi
  - Eukaryotes/Protists
- Sample
- Alignment viewer
- About

☑ Compact format

@fbreitw, 2016

Select sample Case05

Filter taxa Homo sapiens | artificial sequences | unclassified | Mammalia

Figure | Table | Text

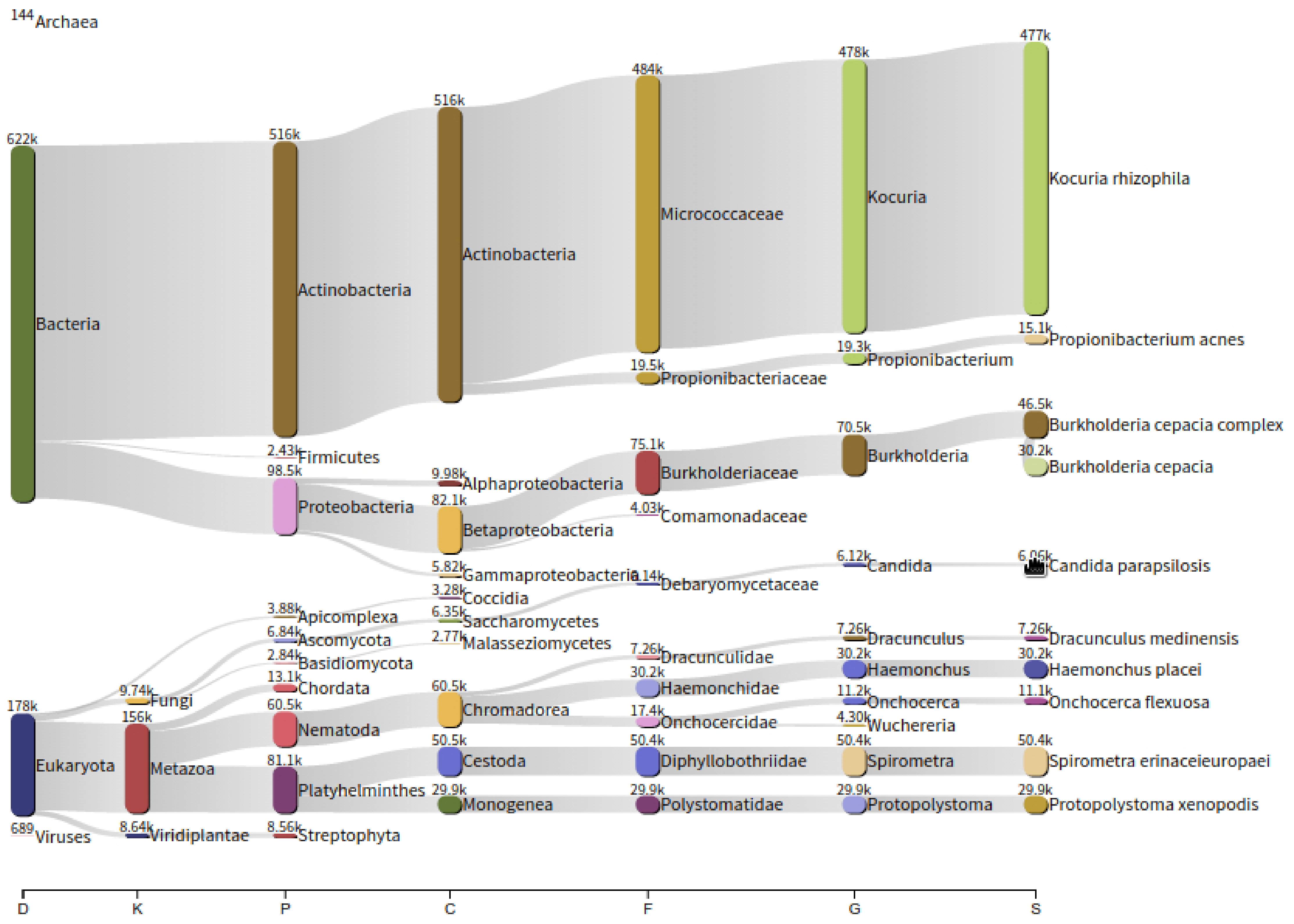

Save Network

Across samples | Figure options

Hover over a node, or click a row in the table, to see the abundance of the taxon in other samples.

cellular organisms>Eukaryota>Opisthokonta>Fungi>Dikarya>Ascomycota>saccharomycet

Candida parapsilosis

Taxonomy rank **Species**, ID 5480. Links: NCBI Taxonomy, Assemblies. Search PubMed, Google or Google Scholar.

Number of reads across all samples

PDF

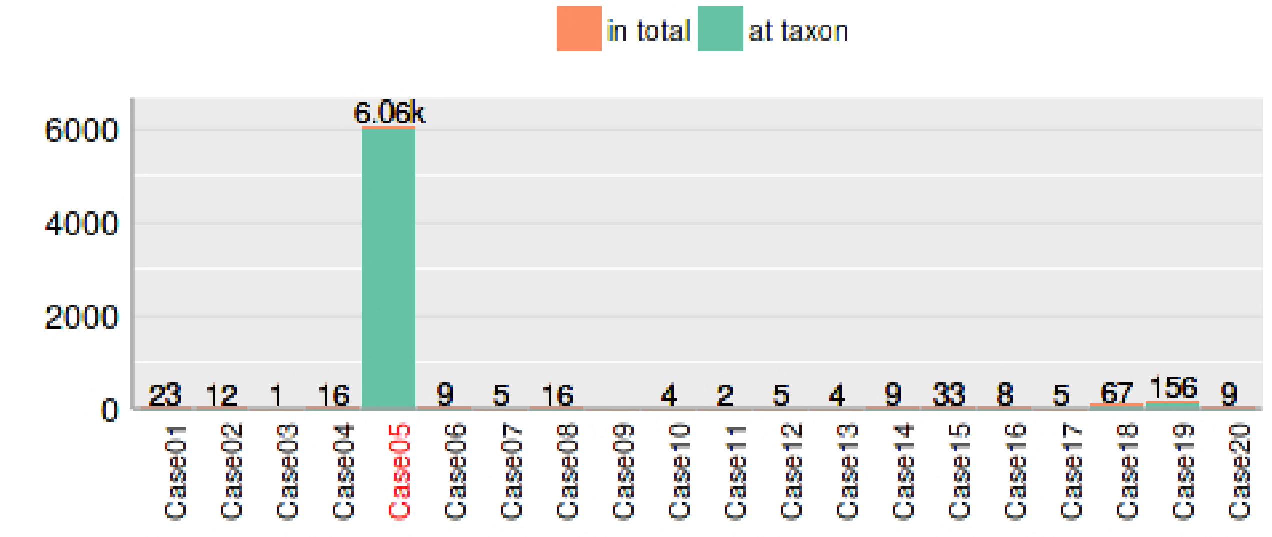

Percent of reads (excluding filtered clades)

PDF

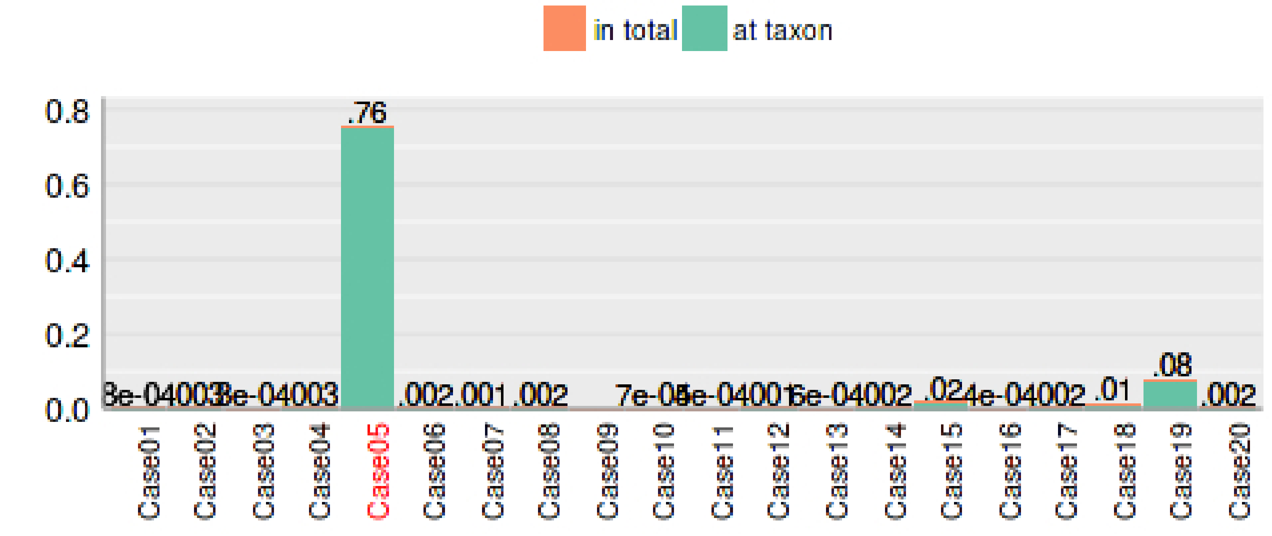

Supplement: Supplement 1 [file iovs-58-14-62_s01.pdf]
