# Supplementary material for: Identifying Corneal Infections in Formalin-Fixed Specimens Using Next Generation Sequencing
Source: Invest Ophthalmol Vis Sci. 2018 Jan;59(1):280–8. doi: 10.1167/iovs.17-21617 (PMC5770184; doi:10.1167/iovs.17-21617)
Supplement: Supplement 2 [file iovs-58-14-62_s02.pdf]

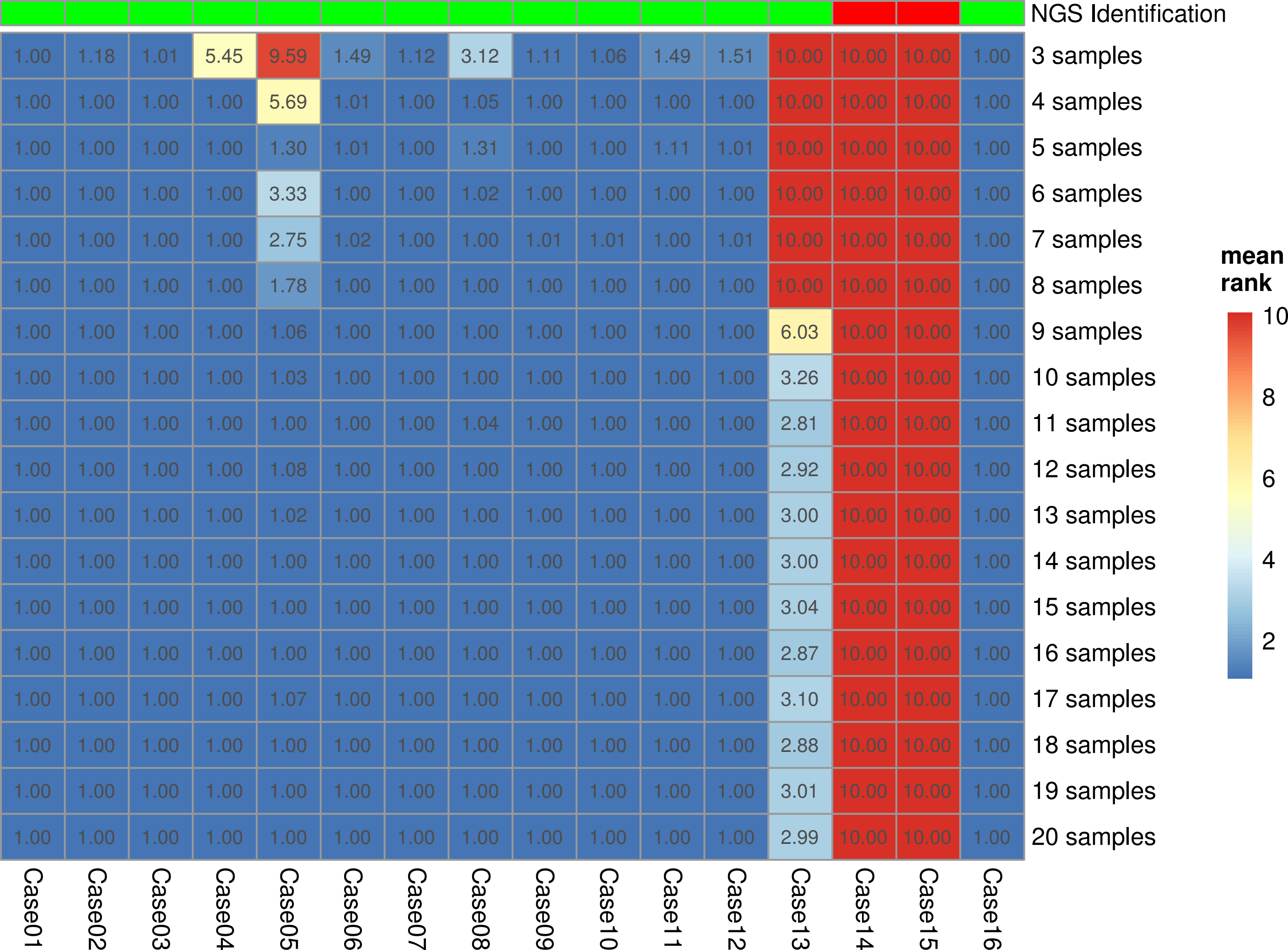
NGS Identification
mean rank
10
8
6
4
2
3 samples
4 samples
5 samples
6 samples
7 samples
8 samples
9 samples
10 samples
11 samples
12 samples
13 samples
14 samples
15 samples
16 samples
17 samples
18 samples
19 samples
20 samples
Case01 Case02 Case03 Case04 Case05 Case06 Case07 Case08 Case09 Case10 Case11 Case12 Case13 Case14 Case15 Case16
1.00 1.18 1.01 5.45 9.59 1.49 1.12 3.12 1.11 1.06 1.49 1.51 10.00 10.00 10.00 1.00
1.00 1.00 1.00 1.00 5.69 1.01 1.00 1.05 1.00 1.00 1.00 1.00 10.00 10.00 10.00 1.00
1.00 1.00 1.00 1.00 1.30 1.01 1.00 1.31 1.00 1.00 1.11 1.01 10.00 10.00 10.00 1.00
1.00 1.00 1.00 1.00 3.33 1.00 1.00 1.02 1.00 1.00 1.00 1.00 10.00 10.00 10.00 1.00
1.00 1.00 1.00 1.00 2.75 1.02 1.00 1.00 1.01 1.01 1.00 1.01 10.00 10.00 10.00 1.00
1.00 1.00 1.00 1.00 1.78 1.00 1.00 1.00 1.00 1.00 1.00 1.00 10.00 10.00 10.00 1.00
1.00 1.00 1.00 1.00 1.06 1.00 1.00 1.00 1.00 1.00 1.00 1.00 6.03 10.00 10.00 1.00
1.00 1.00 1.00 1.00 1.03 1.00 1.00 1.00 1.00 1.00 1.00 1.00 3.26 10.00 10.00 1.00
1.00 1.00 1.00 1.00 1.00 1.00 1.00 1.04 1.00 1.00 1.00 1.00 2.81 10.00 10.00 1.00
1.00 1.00 1.00 1.00 1.08 1.00 1.00 1.00 1.00 1.00 1.00 1.00 2.92 10.00 10.00 1.00
1.00 1.00 1.00 1.00 1.02 1.00 1.00 1.00 1.00 1.00 1.00 1.00 3.00 10.00 10.00 1.00
1.00 1.00 1.00 1.00 1.00 1.00 1.00 1.00 1.00 1.00 1.00 1.00 3.00 10.00 10.00 1.00
1.00 1.00 1.00 1.00 1.00 1.00 1.00 1.00 1.00 1.00 1.00 1.00 3.04 10.00 10.00 1.00
1.00 1.00 1.00 1.00 1.00 1.00 1.00 1.00 1.00 1.00 1.00 1.00 2.87 10.00 10.00 1.00
1.00 1.00 1.00 1.00 1.07 1.00 1.00 1.00 1.00 1.00 1.00 1.00 3.10 10.00 10.00 1.00
1.00 1.00 1.00 1.00 1.00 1.00 1.00 1.00 1.00 1.00 1.00 1.00 2.88 10.00 10.00 1.00
1.00 1.00 1.00 1.00 1.00 1.00 1.00 1.00 1.00 1.00 1.00 1.00 3.01 10.00 10.00 1.00
1.00 1.00 1.00 1.00 1.00 1.00 1.00 1.00 1.00 1.00 1.00 1.00 2.99 10.00 10.00 1.00
