# Supplementary figures and images for: Identifying Corneal Infections in Formalin-Fixed Specimens Using Next Generation Sequencing
Source: Invest Ophthalmol Vis Sci. 2018 Jan;59(1):280–8. doi: 10.1167/iovs.17-21617 (PMC5770184; doi:10.1167/iovs.17-21617)

## Supplementary Figure 3

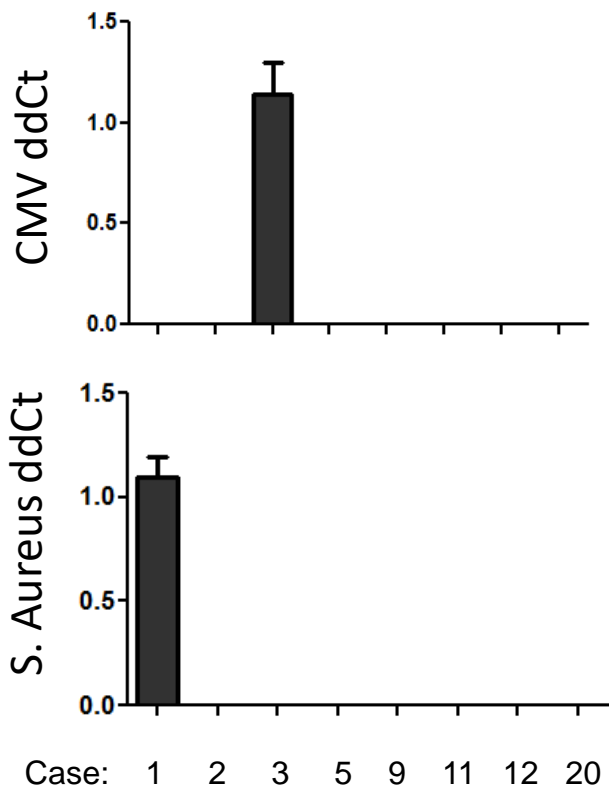

Supplement: Supplement 3 [file iovs-58-14-62_s03.pdf]
