# Supplementary material for: Identifying Corneal Infections in Formalin-Fixed Specimens Using Next Generation Sequencing
Source: Invest Ophthalmol Vis Sci. 2018 Jan;59(1):280–8. doi: 10.1167/iovs.17-21617 (PMC5770184; doi:10.1167/iovs.17-21617)
Supplement: Supplement 4 [file iovs-58-14-62_s04.pdf]

Supplementary Table S1. Sequence reads numbers from initial Kraken analysis

(Shading of each cell corresponds to the relative abundance of DNA reads)

Supplementary Table S2. Z-scores from Centrifuge analysis

(Cells with the highest the z-score in each case are shaded)

Supplementary Table S3. Sequence reads numbers from Centrifuge analysis

(Shading of each cell corresponds to the relative abundance of DNA reads)

Supplementary Table S4. Fresh corneas divided and processed in sterile fashion or as routine formalin fixed paraffin embedded (FFPE) samples.

(Red/Italicized lines are Kraken reads from the eukaryotic pathogen database)

Supplementary Figure S1. Illustration of Pavian Output

The sample view in Pavian presents classifications of one sample in a taxonomical Sankey diagram from the domain- to the species-level. The height of the bars and the flows are proportional to the number of reads. When hovering over a node, two bar plots with absolute and relative number of reads for the

specified taxon in all the other samples are shown (right sidebar). In sample 5, Candida parapsiolosis was our candidate. While this may not be immediately apparent from the classification overview (Sankey diagram on the left), this species is not detected in other sample, and thus unlikely to be due to contamination.

Supplementary Figure S2. Stability of z-score ranking of Centrifuge identifications

The heatmap shows average ranks of the pathogens in cases 1-16 when using bootstrap selection of between two and 19 other samples as backgrounds. Methodology: For each of the 16 cases, we randomly sampled with replacement from two to 19 of the other cases (including negative controls), and calculated the z-score rank for each microbial species identified in that set. For each number of cases we recorded the average rank of the true positive pathogen (found in cases 1-13 and 16 with Centrifuge, see Table 1) over 1000 random pairings. Average ranks over 10 are shown as 10.0.

Supplementary Figure S3. Quantitative PCR analysis of CMV and S. aureus in selected samples

Cases in which CMV or S. aureus was detected by either clinical testing or NGS, along with several additional randomly chosen samples, were examined using qPCR for the presence of DNA from these organisms using previously published primers.
